# Interleukin-36β provides protection against HSV-1 infection, but does not modulate initiation of adaptive immune responses

**DOI:** 10.1038/s41598-017-05363-4

**Published:** 2017-07-19

**Authors:** Katelynn A. Milora, Siva R. Uppalapati, Julio C. Sanmiguel, Wei Zou, Liselotte E. Jensen

**Affiliations:** 1 Department of Microbiology and Immunology, Temple University Lewis Katz School of Medicine, Philadelphia, Pennsylvania USA; 2 Horae Gene Therapy Center, University of Massachusetts Medical School, Worcester, Massachusetts, USA; 3 Schepens Eye Research Institute – MEEI, Harvard Medical School, Boston, Massachusetts, USA; 4 Department of Pharmacology, University of Pennsylvania, Philadelphia, Pennsylvania USA; 5 ProMetic, Washington DC, USA; 6 Fox Chase Cancer Center, Philadelphia, Pennsylvania USA

## Abstract

Interleukin-36 (IL-36) represents three cytokines, IL-36α, IL-36β and IL-36γ, which bind to the same receptor, IL-1RL2; however, their physiological function(s) remain poorly understood. Here, the role of IL-36 in immunity against HSV-1 was examined using the flank skin infection mouse model. Expression analyses revealed increased levels of IL-36α and IL-36β mRNA in infected skin, while constitutive IL-36γ levels remained largely unchanged. In human keratinocytes, IL-36α mRNA was induced by HSV-1, while IL-1β and TNFα increased all three IL-36 mRNAs. The dominant alternative splice variant of human IL-36β mRNA was isoform 2, which is the ortholog of the known mouse IL-36β mRNA. Mice deficient in IL-36β, but not IL-36α or IL-36γ, succumbed more frequently to HSV-1 infection than wild type mice. Furthermore, IL-36β^−/−^ mice developed larger zosteriform skin lesions along infected neurons. Levels of HSV-1 specific antibodies, CD8^+^ cells and IFNγ-producing CD4^+^ cells were statistically equal in wild type and IL-36β^−/−^ mice, suggesting similar initiation of adaptive immunity in the two strains. This correlated with the time at which HSV-1 genome and mRNA levels in primary skin lesions started to decline in both wild type and IL-36β^−/−^ mice. Our data indicate that IL-36β has previously unrecognized functions protective against HSV-1 infection.

## Introduction

Herpes simplex virus-1 (HSV-1) is a common human pathogen that is estimated to be present in up to 90% of the adult population^1^. The virus establishes incurable latent infections in neurons and active disease in the skin or mucosa is triggered by, for example, stress and immune suppression. During active disease the virus proliferates in epithelial cells leading to infectious viral shedding, tissue damage, and formation of vesicles and lesions^1^. In healthy individuals, lesions heal within 1–3 weeks and complications are rarely seen. However, in immune compromised patients and neonates the virus may disseminate to the brain, lung and/or liver with potentially fatal outcomes^2–6^.

T-cells, NK-cells and antibodies are critically involved in restricting local viral replication and dissemination to other sites^1, 7, 8^. However, like most pathogens, HSV-1 has developed immune evasion strategies to prevent or delay detection by the host^9^. One such mechanism involves retention of the pro-inflammatory cytokine interleukin-1β (IL-1β) within infected cells^10, 11^. Interestingly, HSV-1 infected skin keratinocytes can still release IL-1α; a response which appears to promote leukocyte recruitment to infected cells and provides protection from viral dissemination^12^. IL-1α and IL-1β are well known to have pleiotropic effects upon the immune system^13^. For example, they promote inflammation, fever and adaptive immune responses by acting through the same receptor, IL-1 receptor type I (IL-1R1)^13^.

The IL-36 cytokines (IL-36α, IL-36β and IL-36γ) were discovered more than 15 years ago and were, due to 12–50% sequence homology, immediately recognized as related to IL-1α and IL-1β^14, 15^. They were momentarily named IL-1 family members 6, 8 and 9 (IL-1F6, IL-1F8 and IL-1F9), but later renamed under the common name IL-36, in part due to their common use of the IL-1R1 related receptor, IL-1 receptor like 2, IL-1RL2^16–18^, also commonly known as IL-36R. The earlier nomenclature remains unchanged for the mouse genes. The proximity of the genes on chromosome 2 in both humans and mice led to the hypothesis that they arose by gene duplication^14, 15^.

**Figure 1:**
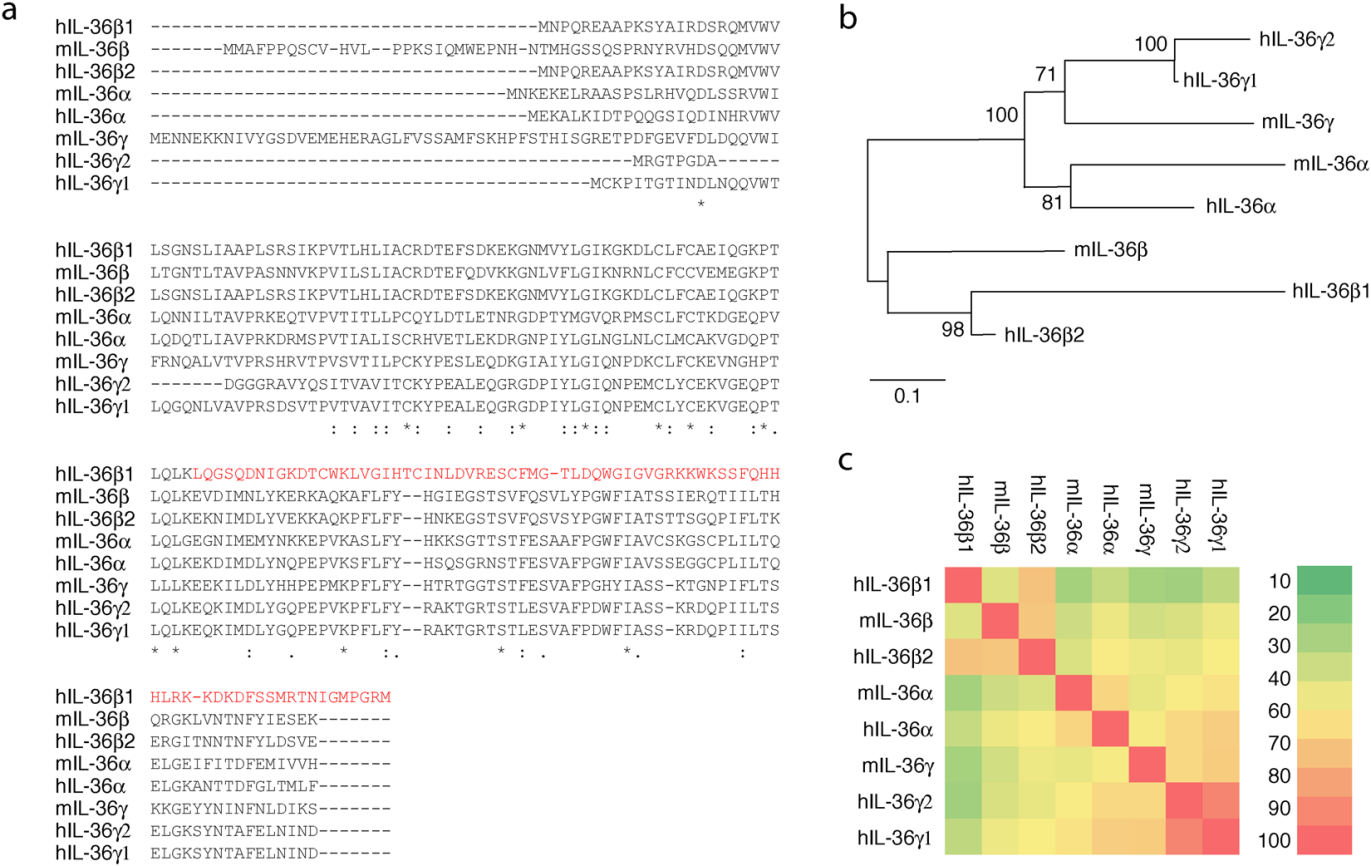
Protein sequence alignment of human and mouse IL-36 cytokines. (**a**) Human (h) and mouse (m) IL-36 cytokines were aligned using Clustal omega. *conserved residues: Gonnet PAM 250 matrix score > 0.5, 0 < Gonnet PAM 250 matrix score ≤ 0.5. Amino acid sequence of human IL-36β isoform 1, which diverge from the other family members, is shown in red. (**b**) Neighbor-joining phylogenetic tree showing relationships among IL-36 cytokines. The optimal tree with the sum of branch length = 2.69 is shown. The percentage of replicate trees in which the associated taxa clustered together in the bootstrap test (1000 replicates) are shown next to the branches. The tree is drawn to scale, with branch lengths in the same units as those of the evolutionary distances used to infer the phylogenetic tree. The evolutionary distances were computed using the Poisson correction method and are in the units of the number of amino acid substitutions per site. (**c**) Heat map showing percentage similarity between the human and mouse IL-36 cytokines.

In both humans and mice there are three genes encoding IL-36α, IL-36β and IL-36γ, respectively. At the amino acid level, these proteins share approximately 15–85% sequence similarity (Fig. 1). Based on human cDNA sequencing, two alternative splice variants of IL-36γ have been identified and entered in GenBank. Compared to IL-36γ isoform 1, isoform 2 lacks a small segment near the N-terminus (Fig. 1) due to the omission of an exon. Humans may also have two splice variants of IL-36β (Fig. 1) with isoform 2 being most homologous to IL-36α and IL-36γ (Fig. 1). Human IL-36β isoform 2 also represents the ortholog of mouse IL-36β (Fig. 1). IL-36β isoform 1 arises from the alternative use of 2 exons down-stream of the most 3′ exon utilized by isoform 2. Consequently, the C-terminal half of IL-36β isoform 1 is distinctive from the other known IL-36 proteins (Fig. 1a, highlighted in red).

During *in vivo* viral infections, IL-36 expression is induced in epithelial cells by HSV-1 (possibly IL-36α)^14^, rhinovirus (IL-36γ)^19^ and influenza (IL-36α)^20^. Further studies have found that expression of IL-36α and IL-36γ mRNAs is increased in lung and skin epithelial cells by a range of TLR ligands, e.g., flagellin, double stranded RNA, lipopolysaccharide (LPS), lipoprotein (FSL-1) and zymosan^21–23^. LPS has been reported to also activate IL-36α and IL-36β expression in monocytes^15^. Expression of IL-36γ in neutrophils has been reported as well^24^; however, neutrophils recruited to the skin in patients with the inflammatory condition generalized pustular psoriasis do not express IL-36γ^25^. In the central nervous system, IL-36β can be expressed by neurons and glia cells^26^.

The IL-1 and IL-36 cytokines lack signal peptides for conventional secretion and are initially synthesized as cytosolic proteins. IL-1β is translated as a 269-amino acid protein that, upon cellular activation, is proteolytically cleaved into the pro-domain (116 amino acids in man) and the functional receptor-binding domain (153 amino acids). This processing promotes extracellular export^13^. The IL-1α mRNA is translated into a 271 amino acid protein, which has functional activity both as full length and processed forms^13^. The IL-36 mRNAs encode significantly shorter 157–183 amino acids proteins (Fig. 1a). Thus, the IL-36 cytokines were initially believed to be synthesized directly as mature proteins, yet without signal peptides^14, 15^. However, the field has struggled with the fact that the recombinant proteins exhibit poor activity, as, for example, noted by Wang *et al*.^26^ and Berglof *et al*.^27^, and required potentially non-physiological concentrations to activate responses in cell culture experiments, e.g., as reported by Towne *et al*.^17^ and Carrier *et al*.^28^. Using bacterially expressed IL-36 cytokines, it was later demonstrated that activity is dramatically improved when a few, very specific, amino acids are removed from the N-terminus^29^. This led to the hypothesis that the IL-36 cytokines require proteolytical processing for activation^29^. Subsequently, *in vitro* cleavage by neutrophil proteases has been demonstrated^30, 31^; however, *in vivo* processing remains to be documented.

The physiological function(s) of the IL-36 cytokines remain poorly understood. Due to increased IL-36α and IL-36γ skin expression and presence of loss-of-function mutations in the natural antagonist, IL-36R antagonist (IL-36Ra), the IL-36 cytokines are believed to play a pathogenic role in plaque and generalized pustular psoriasis^16, 25, 32^. One such mechanism may involve neutrophil recruitment^25, 32–35^. Furthermore, *in vitro* studies suggest that the IL-36 cytokines can stimulate maturation of dendritic cells and downstream polarization of naïve T cells towards IFNγ producing Th1 cells^36–39^. This could suggest a role in immune responses directed against microorganisms; yet, documentation of such a function has remained elusive^36, 40^. Another intriguing aspect of the IL-36 cytokines is the presence of three genes encoding proteins acting on the same receptor as described above.

With the long-term goals of identifying normal physiological functions and the purpose of maintaining the IL-36 gene duplication during evolution, we have started comparing outcomes of challenging IL-36α^−/−^, IL-36β^−/−^ and IL-36γ^−/−^ mice. As part of these studies, we recently reported that inflammation induced by the antiviral drug imiquimod requires IL-36α, but neither IL-36β nor IL-36γ^41^. Interestingly, imiquimod is sometimes used to treat active HSV skin disease caused by strains resistant to acyclovir^42–45^. Extending upon our previous studies, we here examined the role of the individual IL-36 cytokines during HSV-1 infection using the flank skin mouse model as our experimental system. Unexpectedly, we found that mice deficient in IL-36β, but not IL-36α, developed more severe disease; however, IL-36β was not essential for initiation of adaptive immunity against HSV-1. Hence, IL-36β appears to have previously unrecognized functions that protect against the outcome of HSV-1 infection.

## Results

### IL-36β deficient mice have increased mortality following HSV-1 infection

**Figure 2:**
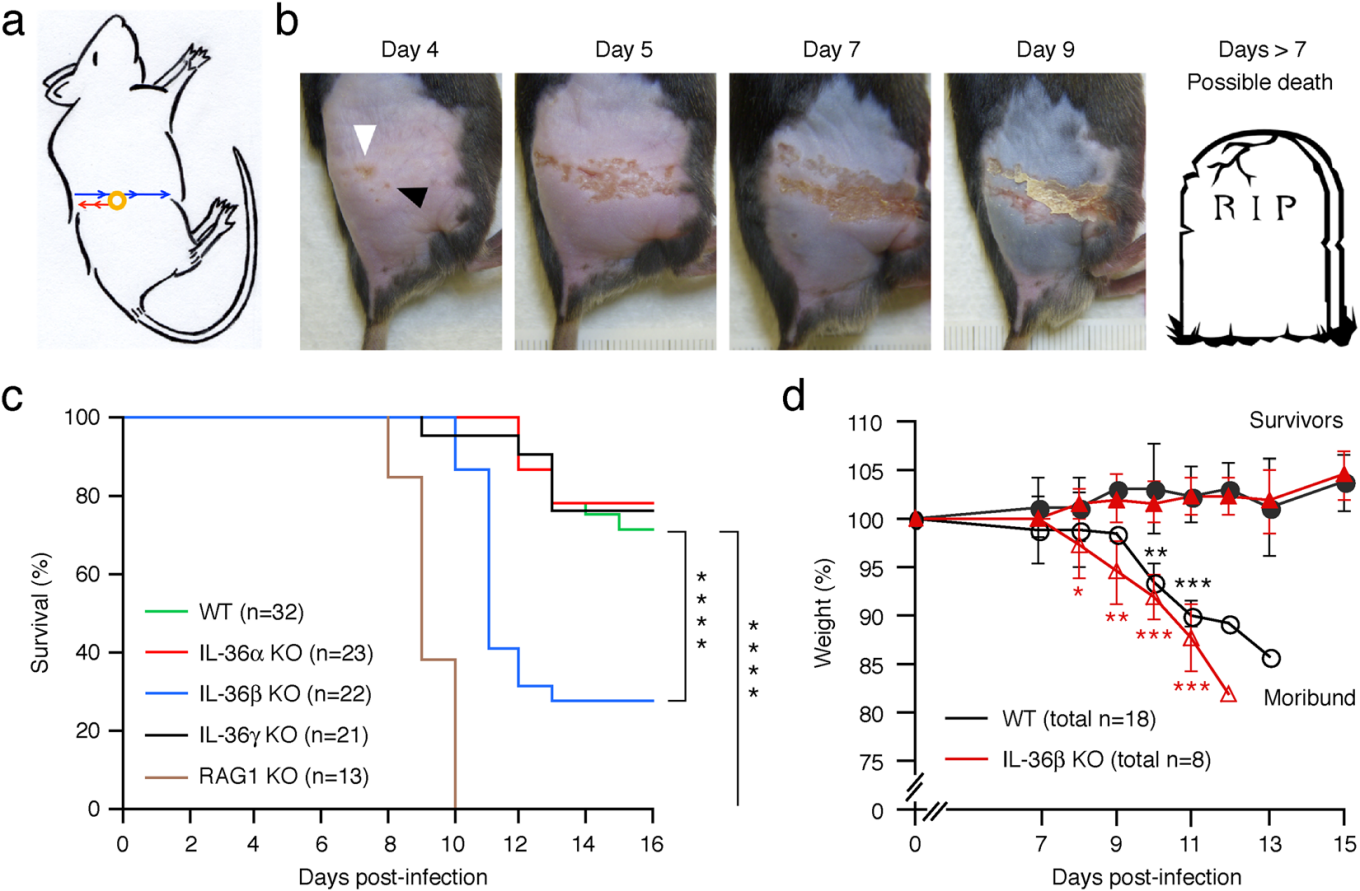
IL-36β, but not IL-36α or IL-36γ, provides protection against lethal outcome of HSV-1 infection. (**a**) Illustration of inoculation site (yellow circle) and directions of retrograde (red arrows) and anterograde (blue arrows) migration of HSV-1 through neurons. (**b**) Progression of disease in wild type mice (C57BL/6) infected with 1.5 × 10^6^ PFU HSV-1 on flank skin (white arrowhead) at day 0 is depicted at the indicated time-points. Black arrowhead points to early secondary lesion appearing along sensory neurons (note the linear progression of the lesions). Ruler used for quantification of lesion sizes visible in day 9 image. Image of gravestone can be found at: https://freeclipartnow.com/holidays/halloween/graveyard/R-I-P-gravestone.jpg.html. (**c**) Wild type (C57BL/6, green), IL-36α^−/−^ (red), IL-36β^−/−^ (blue), IL-36γ^−/−^ (black), and RAG1^−/−^ (brown) mice were infected with 1.5 × 10^6^ PFU HSV-1 on the right flank on day 0, and survival monitored for 16 days. n indicates number of mice per group. Data shown is pooled from 6 independent experiments. Statistical significance was determined using Mantel-Cox and Gehan-Breslow-Wilcoxon tests; ****p < 0.0001. (**d**) Wild type (C57BL/6, n = 18, black lines and symbols) and IL-36β^−/−^ (n = 8, red lines and symbols) mice were infected with HSV-1 as described above. Pooled weight data (means ± SD) of survivors (closed symbols) and mice progressing to become moribund (open symbols) from two independent experiments is shown. *p < 0.05 (comparing survivors to moribund within each strain); **p < 0.01; ***p < 0.001.

Using IL-36α knockout (KO) mice, we previously demonstrated that IL-36α is critical for the skin inflammation induced by the antiviral drug imiquimod^41^. In contrast, IL-36β^−/−^ and IL-36γ^−/−^ mice developed the same degree of skin inflammation as wild type mice^41^. Indirectly, this could suggest a role for one or more IL-36 cytokines in immune responses against viruses. Interestingly, when the IL-36s were discovered it was demonstrated that expression of one, likely IL-36α, was up-regulated in keratinocytes infected with HSV-1^14^; however, the function was not explored. Thus, we examined progression of HSV-1 infection in wild type, IL-36α^−/−^, IL-36β^−/−^, and IL-36γ^−/−^ mice by employing the flank skin HSV mouse model. We have previously reported that the IL-36 knockout (KO) mice have no spontaneous phenotypes precluding this study^41^. In the flank model, HSV-1 enters sensory neurons at the site of primary infection (Fig. 2a, yellow circle). From here it migrates through the neurons to the dorsal root ganglia (Fig. 2a, red arrows), where it can establish latency. Subsequently, viral replication, anterograde migration (Fig. 2a, blue arrows) and shedding leads to formation of secondary zosteriform lesions along the affected neurons (Fig. 2b, days 4–7, note the linear lesion pattern forming 5 days post-infection)^1^. Using this model, we found that IL-36α^−/−^ and IL-36γ^−/−^ mice exhibited the same mortality rate (Fig. 2c, median survival time, >16 days) as wild type mice. In contrast, the IL-36β^−/−^ mice revealed significantly reduced survival (Fig. 2c, median survival time: 11 days). Fatal outcome in both wild type and IL-36β^−/−^ mice was associated with progressive weight loss (Fig. 2d). The increased mortality rate of the IL-36β KO mice strongly suggest an important involvement of IL-36β in controlling the outcome of HSV-1 infection.

### Lethal outcome is associated with bowel dysfunction syndrome in both wild type and IL-36β KO mice

**Figure 3:**
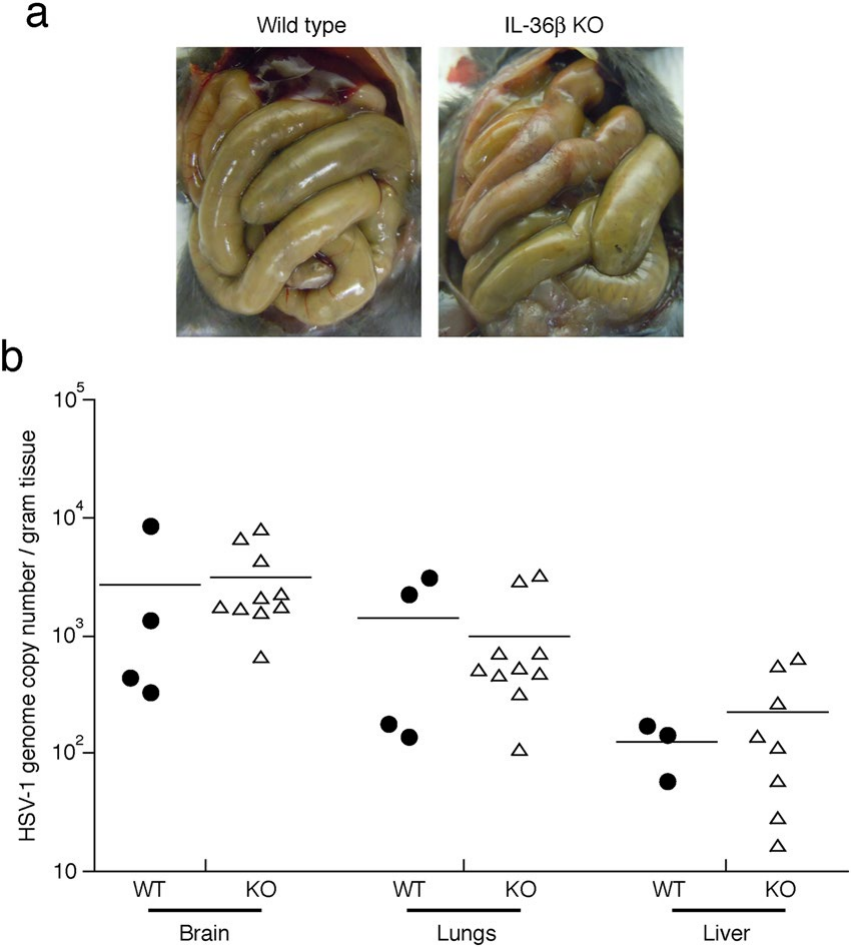
Mortality is associated with viral dissemination to multiple organs. (**a**) Representative images of bowels of moribund wild type and IL-36β^−/−^ (Fig. 2) mice are shown. (**b**) Wild type (black circles) and IL-36β^−/−^ (open triangles) mice were infected with 1.5 × 10^6^ PFU HSV-1 on the right flank. Organs were collected from moribund mice, homogenized and HSV-1 genome copy numbers determined by QPCR. Each symbol represents a single mouse. Data is pooled from two independent experiments. No statistical significant differences were observed between wild type and IL-36β deficient mice.

It has been reported that mice infected vaginally with HSV-2 (strain 333) develop paralysis, constipation and bladder retention^46^. These outcomes could be the result of inflammation causing demyelination in the nervous system^47^. Using the flank model and HSV-1 (strain NS), we previously observed a bowel dysfunction syndrome evident by greatly enlarged colons and small intestines in moribund wild type and IL-1R1 KO mice^12^. Recently, the lethal outcome of vaginal HSV-1 infection in mice was linked to these phenotypes, as the virus spreads through the nervous system resulting in inflammation-mediated damage to the enteric neurons and toxic megacolon (constipation)^48^. Here we found that moribund wild type and IL-36β KO mice exhibited greatly enlarged small and large intestines (Fig. 3a). This bowel dysfunction syndrome likely explains the lethal outcome in the flank model.

### Viral DNA is present in brain, liver and lungs in moribund wild type and IL-36β KO mice

In humans HSV can disseminate to internal organs such as the brain, liver and lungs, where it causes organ damage leading to significant morbidity and mortality^2–6^. We previously showed that in the HSV-1 flank model the virus disseminates to internal organs such as the brain, liver and lungs^12^. Furthermore, we reported that IL-1R1 KO mice exhibit an increased mortality following HSV-1 infection^12^ similar to that reported here for IL-36β (Fig. 2c); yet levels of viral genomic DNA in internal organs from moribund IL-1R1 deficient mice did not differ from those of wild type moribund mice^12^. Upon analyses of organs from moribund IL-36β KO mice, we found that HSV-1 genome copy numbers were similar to those found in moribund wild type mice (Fig. 3b). This could suggest that HSV-1 disseminates to the same organs in wild type and IL-36β KO mice.

### IL-36β KO mice develop antibodies against HSV-1 at the same time as wild type mice

**Figure 4:**
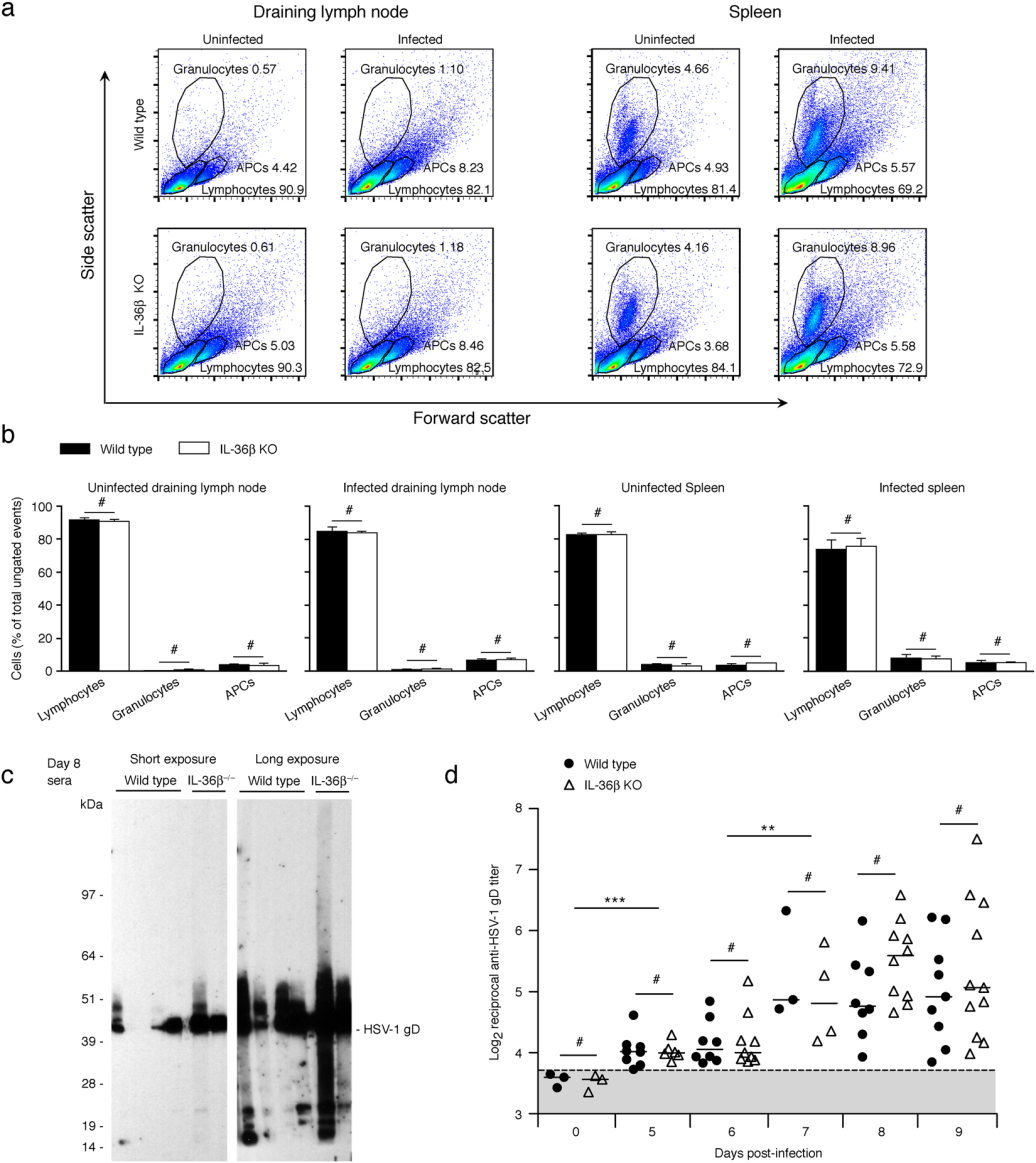
IL-36β^−/−^ mice develop antibodies against HSV-1 at the same time as wild type mice. (**a**) Wild type and IL-36β^−/−^ mice were infected with HSV-1 (n = 5 per group) or left uninfected (n = 2–3 per group). Cells were isolated from spleen and draining inguinal lymph nodes 6 days post-infection and analyzed by flow cytometry. Granulocyte, lymphocyte, and antigen presenting cell (APCs) populations were quantified via gates depicted on representative images of forward scatter and side scatter. (**b**) Graphic representation of data (means ± SD) from **a**. ^#^ *p* > 0.05. Representative experiment of 3. (**c** and **d**) Wild type (as labeled in **c**, black circles in **d**) and IL-36β^−/−^ (as labeled in **c**, open triangles in **d**) mice were infected with HSV-1 and serum collected at the indicated time-points. (**c**) Reactivity of mouse sera against HSV-1 infected HaCaT protein lysate was examined by Western blotting. The HaCaT protein lysate was separated by SDS-PAGE using a gel with a single approximately 7 cm wide well. After transfer to a PVDF membrane, multiple strips approximately 2 mm wide were cut and used for incubation with individual sera. Approximate positions of protein markers are shown. (**d**) Reactivity of mouse sera against HSV-1 gD were determined by direct ELISA. Log_2_ reciprocal values of end-point titers are depicted. Gray box indicates background signal against trace *E. coli* proteins. No statistically significant differences were observed between wild type and IL-36β deficient mice (#) despite significant increases in anti-gD titers in both strains (**p < 0.01 (compared as indicated); ***p < 0.001). Each symbol represents a single mouse.

The adaptive immune system is essential for immunity against HSV^1, 7, 8^. *In vitro* studies have demonstrated that IL-36 up-regulates expression of MHC class II and CD83 on dendritic cells^37, 39, 49^; thus, IL-36 may promote the development of adaptive immune responses *in vivo*. In our HSV-1 flank model, mice deficient in B and T cells (RAG1 KO mice) had a median survival time of 9 days (Fig. 2c). Given the timing of death observed with the RAG1 and IL-36β (Fig. 2c) KO mice, we hypothesized that IL-36β protects against HSV-1 by promoting the development of adaptive immunity towards the virus. Initial analyses of leukocyte populations in spleens and draining inguinal lymph nodes revealed no significant differences in the proportions of granulocytes, antigen presenting cells and lymphocytes in wild type and IL-36β KO mice in the presence or absence of infection (Fig. 4a,b).

In mice, antibodies can protect against lethal outcome of HSV infection^50^, including that caused by the NS strain used here^51, 52^. Hence, we examined the progressive development of HSV-1 antibodies in wild type and IL-36β KO mice. HSV-1 gD has been reported to be the major antigen towards which early antibodies are directed during a primary HSV infection (see ref. 51 for refs.). In agreement with this, we observed potent reactivity towards an approximately 43 kDa protein band likely representing HSV-1 gD (Fig. 4c). Similar banding patterns were observed in wild type and IL-36β KO mice (Fig. 4c). Quantitative analyses (Fig. 4d) revealed appearance of increased antibody levels already at day 5 in both wild type and IL-36β KO mice (Fig. 4d). These levels increased up to day 9 when RAG1^−/−^ died; however, no statistically significant differences between wild type and IL-36β KO mice were detected (Fig. 4d). This outcome demonstrates that IL-36β plays no role in initiating a primary antibody response in the present model system.

### Wild type and IL-36β KO mice develop gB(498–505) specific CD8^+^ cells at the same time

**Figure 5:**
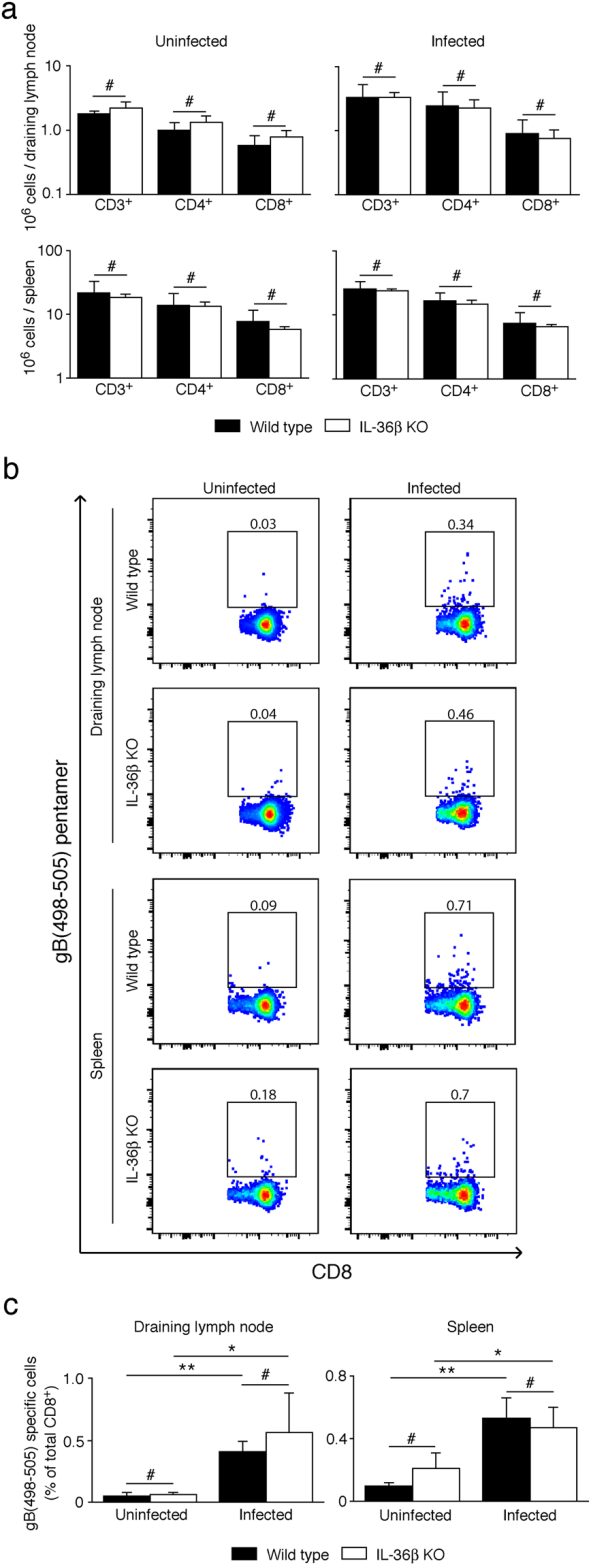
Wild type and IL-36β KO mice have equal numbers of early HSV-1 gB(498-505) specific CD8^+^ cells. Wild type and IL-36β KO mice were infected with HSV-1 (n = 5 per group) as described in Fig. 2 or left uninfected (n = 2–3 per group). Cells were isolated from spleen and draining inguinal lymph nodes 6 days post-infection and analyzed by flow cytometry. (**a**) Total number of CD3, CD4 or CD8 positive cells per organs are shown. (**b**) Cells were examined following initial gating of the CD3^+^ population. The square gates identify the gB(498–505)/CD8 double positive cells. Numbers above the gates indicate percentage gated cells of all CD8^+^ cells. (**c**) Graphic representation of gB(498–505) specific CD8^+^ T cells (means ± SD) quantified in **b**. One representative experiment of 3 is shown. ^#^ *p* > 0.05; *p < 0.05, **p < 0.01.

CD8^+^ cells can kill HSV-1 infected cells^8^, and start clearing the primary HSV-1 infection in the mouse flank skin model as early as day 5 post-infection^53^. Since IL-36β deficient mice developed antibodies at the same time as wild type mice, we next examined their ability to develop HSV-1 specific CD8^+^ cells. Overall levels of the major T cell populations were indistinguishable in wild type and IL-36β KO spleens and draining inguinal lymph nodes in both uninfected and infected mice (Fig. 5a). In mice, the majority of HSV-1 specific CD8^+^ cells are directed against the epitope gB(498–505) comprising the sequence SSIEFARL^54^. To address if the initiation of the development of these cells requires IL-36β, we examined levels of HSV-1 gB(498-505) specific cells 6 days post-infection. Since this is a very early time point, numbers of these cells were expected to be low. In both wild type and IL-36β KO mice, the percentage of CD8^+^ cells specific to gB(498-505) increased significantly in both the draining inguinal lymph nodes and the spleens post-infection (Fig. 5b,c); however, no significant differences between wild type and IL-36β KO mice were detected (Fig. 5b,c). This suggests that IL-36β does not regulate processes required for initiation of CD8 mediated cellular immunity.

### HSV-1 replication appears to progress similarly at the site of primary infection in wild type and IL-36β^−/−^ mice

**Figure 6:**
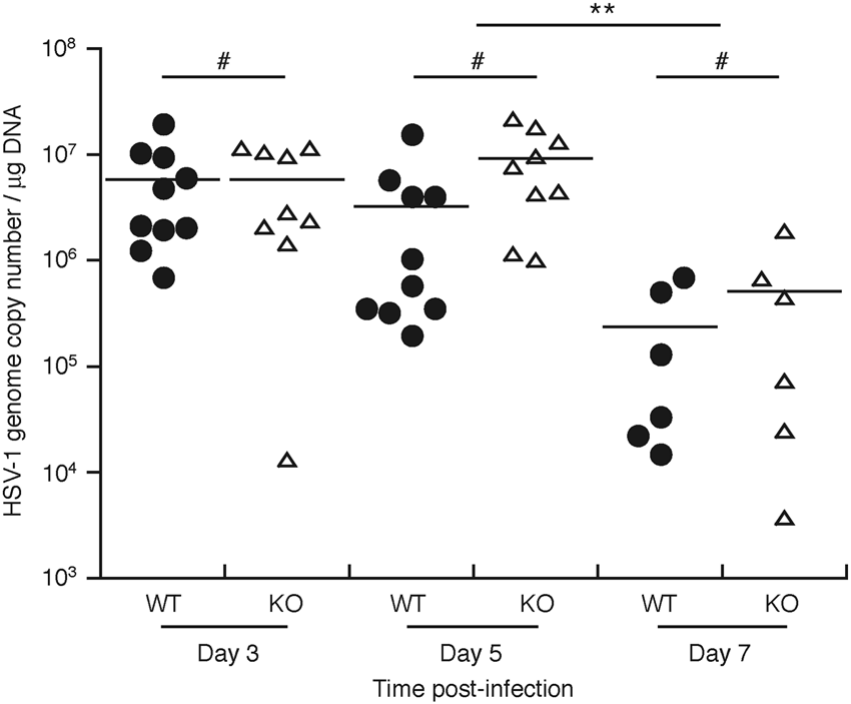
Primary skin infection site viral genome copy numbers are indistinguishable in wild type and IL-36β KO mice. Wild type (black circles) and IL-36β^−/−^ (open triangles) mice were infected with HSV-1 on the flank skin as described in Fig. 2. The center of primary infection sites were collected with 4 mm punch biopsy tools at the indicated time-points. Viral genome copy numbers were determined by QPCR. Each symbol represents a single mouse. **p < 0.01; ^#^ *p* > 0.05.

The similar induction of adaptive immune responses in wild type and IL-36β KO mice (Figs 4, 5) could suggest comparative abilities to clear viral infection. Analyses of HSV-1 genome copy numbers (Fig. 6) in primary infections sites were found to be not statistically significantly different in the wild type and IL-36β KO mice on days 3 and 5. This could suggest that HSV-1 replicates in the skin at similar rates in both strains. Furthermore, the viral genome copy numbers started to decrease at the same time, i.e., leading to a significant reduction in HSV-1 genome copy numbers from day 5 to day 7 (Fig. 6). The timing of this decrease correlated with induction of early HSV-1 specific antibodies (Fig. 4). This provides further evidence that adaptive immune responses aimed at clearing the skin infection are equally well initiated in the wild type and IL-36β KO mice.

### IL-36β deficient mice develop more severe secondary zosteriform skin lesions

**Figure 7:**
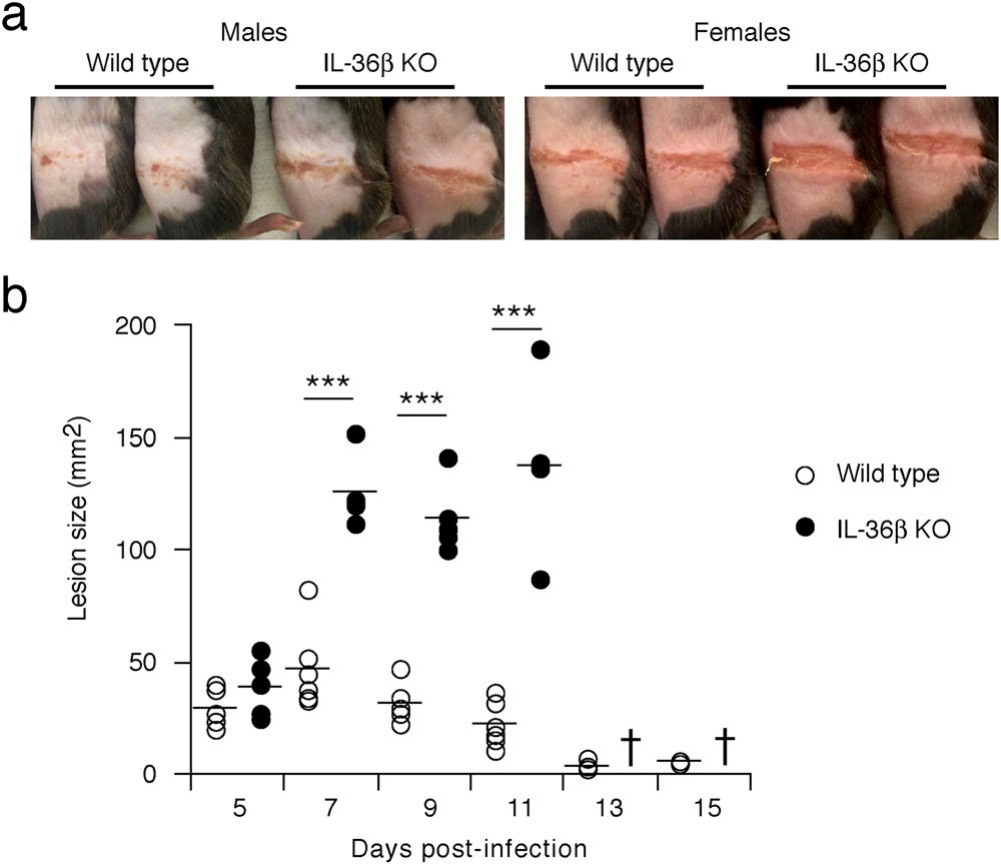
IL-36β deficient mice develop more severe secondary zosteriform skin lesions. (**a**) Wild type and IL-36β KO mice were infected with HSV-1 as described in Fig. 2. Male and female wild type and IL-36β KO mice were anesthetized and photographed at day 6 post-infection. (**b**) Wild type (open circles, n = 6) and IL-36β KO (black circles, n = 5) mice were infected with HSV-1 as described in Fig. 2 and progression of secondary zosteriform lesions photo-documented for 15 days as illustrated in **a** and Fig. 2. Lesions were sized using ImageJ. †, all mice dead; ***p < 0.001.

An interesting aspect of the flank model of HSV-1 skin infection is the re-dissemination of the virus from the dorsal root ganglion, through the sensory neurons, to the skin, where it causes the formation of secondary zosteriform lesions along the dermatome several days after primary infection (Fig. 2a,b). In IL-1R1 KO mice these secondary lesions appear at the same time as in wild type mice^12^. Furthermore, the lesions progress in size in a similar manner^12^. Interestingly, in the present study we found that these zosteriform lesions emerged approximately at the same time in wild type and IL-36β KO mice (Fig. 7, day 5) suggesting that the virus migrates through the neurons at the same rate. However, the secondary lesions progressed to become significantly larger in IL-36β KO mice than in wild type (Fig. 7, days ≥ 6). This may implicate IL-36β in mechanisms aimed at limiting wound progression as the virus reemerges from the neurons.

### Levels of IFNγ-producing CD4^+^ cells are similar in wild type and IL-36β KO zosteriform lesions

**Figure 8:**
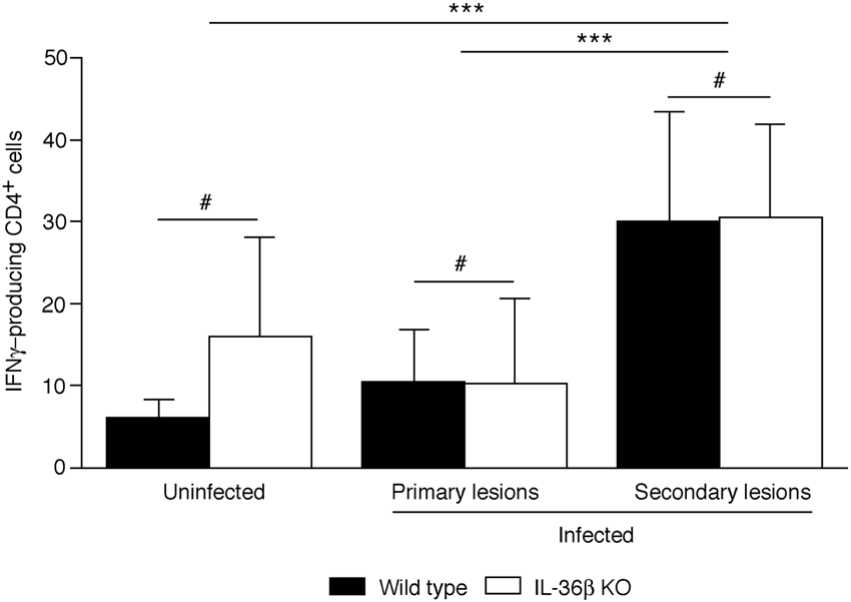
Number of IFNγ-producing CD4^+^ cells are similar in wild type and IL-36β KO zosteriform lesions. Wild type (black bars) and IL-36β KO (open bars) mice were infected with HSV-1 (n = 10 per group) or left uninfected (n = 3–5 per group). Skin was collected 6 days post-infection. Primary lesion sites were collected with 8 mm biopsy punches at the center of the initial infection sites. Upper and lower secondary lesions (Fig. 2b) were excised and pooled. Number of IFNγ-producing CD4 + cell were determined using ELISpot assays. Representative data (means ± SD) from one of two experiments are shown. ^#^ *p* > 0.05; *p < 0.05; **p < 0.01.

IFNγ-producing CD4^+^ cells provide protective immunity against HSV infections in peripheral epithelial tissues^55, 56^. Since IL-36 has been shown to promote differentiation of naïve T cells into IFNγ-producing Th1 cells^37, 57^, we hypothesized that the larger zosteriform lesions could be due to reduced levels of these cells in the skin. Significantly increased levels of IFNγ-producing CD4^+^ cells were found in skin surrounding early secondary lesions compared to uninfected and primary infection sites (Fig. 8). However, no statistically significant differences in IFNγ-producing CD4^+^ cell levels were found when comparing wild type to IL-36β KO mice (Fig. 8). This suggests that IL-36β KO mice have an uncompromised capacity to activate and/or recruit IFNγ-producing CD4^+^ cells.

### IL-36 expression is induced during *in vivo* infection in mice

**Figure 9:**
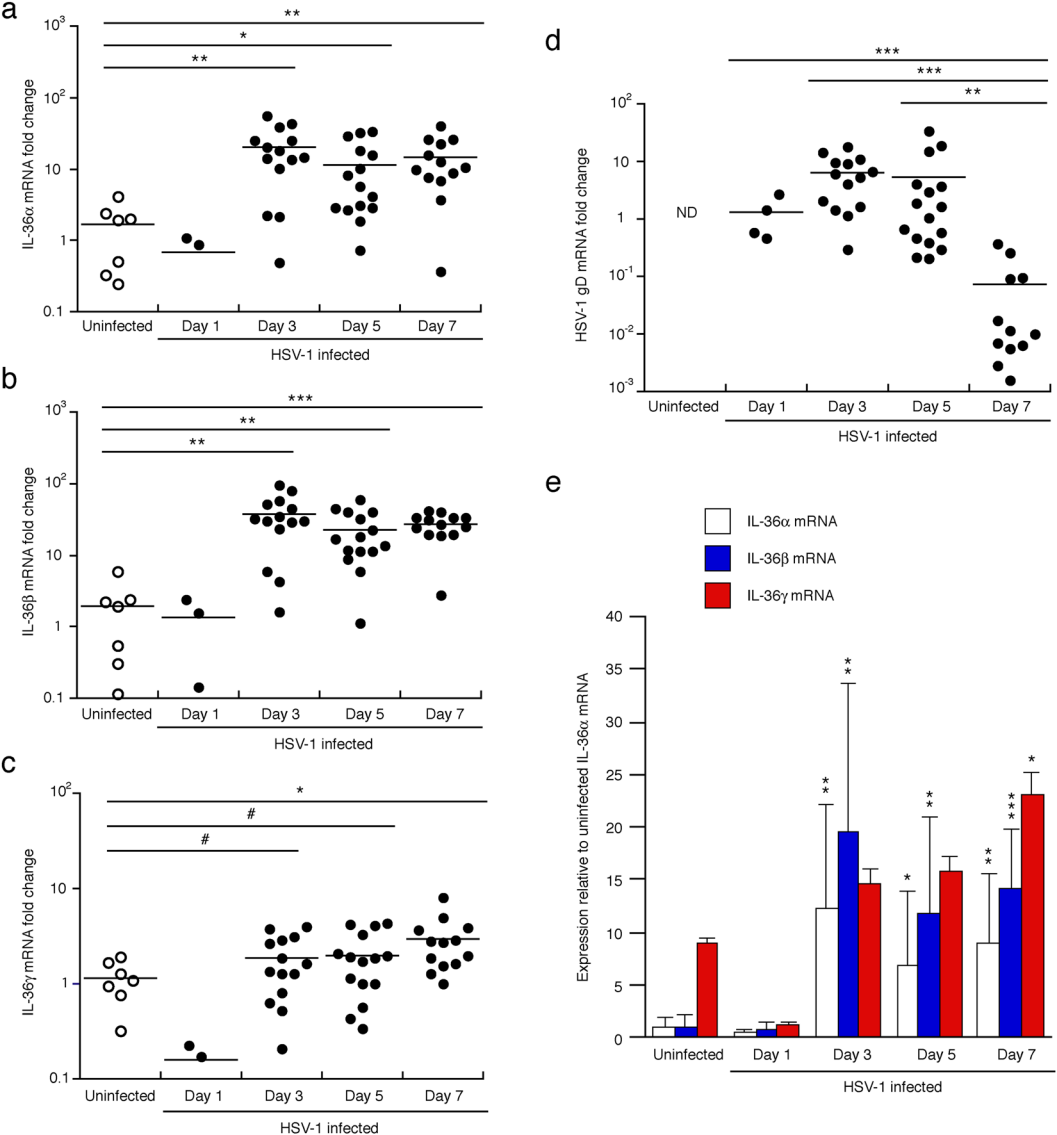
IL-36 mRNA expression is induced *in vivo*. (**a**–**d**) C57BL/6 mice were infected with HSV-1 (black symbols) or left uninfected (open symbols). At the indicated time-points primary or mock lesions were collected at the center using 4 mm punch biopsies. IL-36 (**a**–**c**) and HSV-1 gD (**d**) mRNA levels determined by real-time PCR using GAPDH as the housekeeping gene against which individual mRNA levels were standardized. Fold changes in IL-36 mRNA levels were calculated against uninfected skin. Fold changes in HSV-1 gD mRNA levels were calculated against levels at day 1. Data shown is pooled from three independent experiments. (**e**) IL-36α (open bars), IL-36β (blue bars) and IL-36γ (red bars) mRNA levels were recalculated (data from **a**–**c**) relative to levels of the IL-36α mRNA in uninfected mice. *p < 0.05; **p < 0.01; ***p < 0.001; ND, not detected.

To possibly explain the specific role of IL-36β, but not IL-36α and IL-36γ, in protection against HSV-1 infection (Fig. 2c), we examined *in vivo* IL-36 expression in the skin during HSV-1 infection (Fig. 9a–c). Expression of the IL-36α and IL-36β mRNAs was dramatically increased (approximately 20–40-fold) in primary lesions starting at day 3, and levels remained elevated through day 7 (Fig. 9a,b). In contrast, the IL-36γ was only modestly induced (2-3-fold) by day 7 (Fig. 9c). Induction of the IL-36 mRNAs appeared delayed compared to viral replication as measured through levels of HSV-1 gD mRNA (Fig. 9d). Furthermore, the IL-36 mRNA levels remained elevated by day 7 (Fig. 9a–c), as viral levels decreased (Fig. 9d). The HSV-1 gD mRNA levels, including the observed decrease in the HSV-1 gD mRNA levels at day 7, correlate with the observed HSV-1 DNA genome copy numbers (Fig. 6). This could suggest that the induction of IL-36 expression is secondary to the infection, i.e., not a direct response to HSV-1.

Next the relative expression of the three IL-36 mRNAs was examined (Fig. 9e). Analyses in uninfected skin revealed that the IL-36γ mRNA is constitutively expressed at approximately 10-fold higher levels than the IL-36α and IL-36β mRNAs (Fig. 9e). However, due to the strong induction of the IL-36α and IL-36β mRNAs (Fig. 9a,b), a trend towards the IL-36β mRNA being the dominant form at day 3 may be detected.

### IL-36α, but not IL-36β and IL-36γ, expression is induced in human keratinocytes during *in vitro* HSV-1 infection

**Figure 10:**
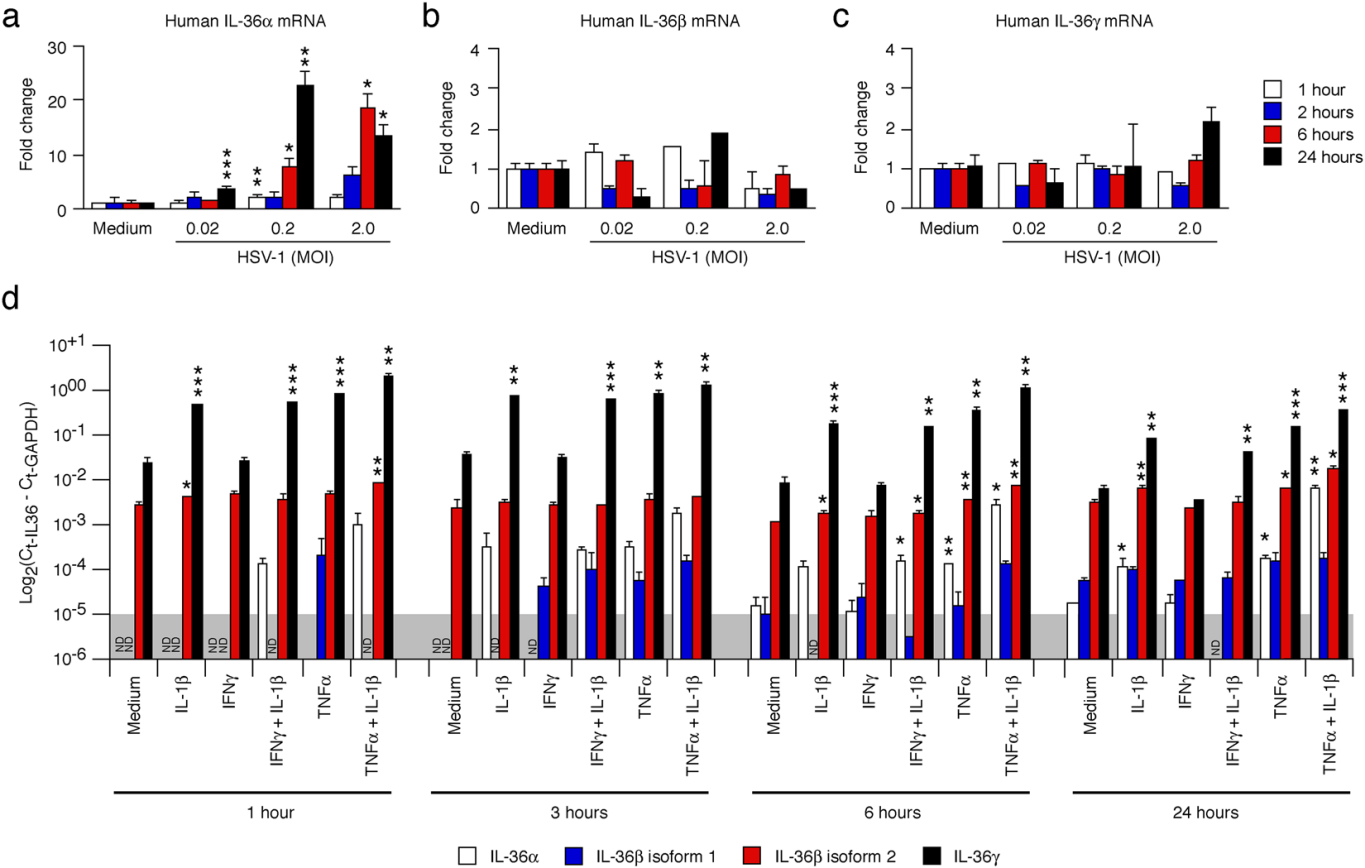
Human IL-36 mRNAs are differentially induced in response to HSV-1 infection and cytokines. Human primary keratinocytes were infected with HSV-1 (**a**–**c**) or treated with medium only, 10 ng/ml IL-1β, 20 ng/ml IFNγ and/or 50 ng/ml TNFα (**d**) as indicated. IL-36α, IL-36β (isoform 1 and 2 in **d**) and IL-36γ mRNA levels were determined by real-time PCR and standardized against GAPDH levels. (**a**–**c**) Fold changes in IL-36 expression were calculated against medium only treated samples at the same time-point. (**d**) Proportional expression of IL-36 levels are graphed as log_2_(C_t-IL-36_−C_t-GAPDH_). Representative data from one of at least three independent experiments is shown as means ± SD. *p < 0.05 (compared to medium only at the same time-point); **p < 0.01; ***p < 0.001.

Previously, we showed that human primary keratinocytes have high levels of pre-formed IL-1α that are released upon HSV-1 infection^12^. In contrast, unstimulated keratinocytes express only very low levels of IL-36β mRNA; however, the expression is increased approximately 10-fold upon poly(I:C) treatment^22^. Here we found that HSV-1 infection induced expression of IL-36α (Fig. 10a). However, we observed no significant increase in the IL-36β and IL-36γ mRNAs (Fig. 10b,c).

### Cytokines induce expression of all three IL-36 mRNAs

The delayed *in vivo* induction of the IL-36 mRNAs could suggest secondary responses. Expression of the IL-36 mRNAs can be induced by cytokines such as IL-1, IFNγ (IL-36β only) and TNFα^21, 28^. We previously showed that these cytokines can induce chemokine expression in keratinocytes in additive and synergistic manners^58^. Here, we found that IL-1β and TNFα significantly induced all three IL-36 mRNAs (Fig. 10d); sometimes with synergistic effects (IL-36α mRNA 24 hours, Fig. 10d). In some (not shown), but not all (Fig. 10d), experiments we observed increased expression of the IL-36β mRNA in response to IFNγ. Overall, the IL-36α and IL-36γ mRNAs appeared the most dramatically induced with IL-36γ exhibiting the highest levels of expression (Fig. 10d).

### IL-36β isoform 2 is the major IL-36β isoform expressed in human keratinocytes

Interestingly, two alternative splice variants of human IL-36β mRNA have been sequenced (Fig. 1). Isoform 2 represents the ortholog of the known mouse IL-36β mRNA (Fig. 1), whereas isoform 1 is substantially distinct from the other IL-36 cytokines (Fig. 1). Isoform specific analyses of human keratinocyte mRNA revealed that the IL-36β isoform 2 mRNA was expressed at dramatically higher levels (>50-fold at all time-points and treatments) than IL-36β isoform 1 (Fig. 10d). This suggests that most of the IL-36β expressed by keratinocytes will have activity similar to those of IL-36α and IL-36γ.

## Discussion

A role for the IL-36s in immunity against microorganisms has been suggested by their induction in epithelial cells in response to several pathogen associated molecular patterns *in vitro* ^21, 22^ as well as HSV-1, rhinovirus and influenza *in vivo* ^14, 19, 20^. However, the physiological functions of the IL-36s during infections remain poorly understood. Using KO mice for each individual IL-36 cytokine, we found that IL-36β, but not IL-36α or IL-36γ, is critically involved in protecting against the outcome of HSV-1 skin infection. We specifically observed that IL-36β KO mice develop larger skin lesions (Fig. 7) and have decreased survival (Fig. 2c). This suggests an essential role for IL-36β in controlling the outcome of the viral infection.

The IL-36s can activate dendritic cells *in vitro* ^36–39, 57^ and *in vivo* in an experimental model of psoriasis, a chronic inflammatory skin condition^59^. This could suggest that the IL-36s play a role in activating the adaptive immune response. Interestingly, we find that IL-36β KO mice start to clear the viral skin infection as quickly as the wild type mice (Fig. 6, day 7; Fig. 9d, day 7). Furthermore, we were unable to detect differences in levels of antibodies (Fig. 4c,d), gB(498-505) specific CD8^+^ (Fig. 5b,c) and IFNγ-producing CD4^+^ cells (Fig. 8) in wild type and IL-36β KO mice. This suggests that IL-36β does not play a significant role in initiating adaptive immunity against the examined immunogens in the presently used model. Since phenotypic differences are observed between wild type and IL-36β KO mice, IL-36β must have other essential physiological functions during HSV-1 infection. Although IL-36β did not contribute to the development of adaptive immunity in the present studies, the IL-36 cytokines may still be involved in these responses. Our initial working hypothesis was that the IL-36 cytokines could overcome HSV-1 immune evasion that inhibits the function of IL-1β^10, 11^ and presumably IL-18 by similarity. IL-1α and IL-33 are related cytokines that are highly expressed in keratinocytes and play a significant role in immunity against HSV infection^12, 60^. The presence of these cytokines could be enough to initiate adaptive immunity in the present HSV infection model. Further studies involving mice unable to signal via all these cytokines will be required to test this possibility. In addition, IL-36β may play a role in recruitment of immune cells (discussed below) and maturation of the immune response. We here examined early immunity against the dominant antibody gD (Fig. 4d) and CD8^+^ gB (Fig. 5b,c) immunogens. However, over time the immune response develops to enhance epitope affinity and diversity. Future analyses of such responses may reveal if IL-36β regulates these processes. Similar analyses of IL-36α and IL-36γ functions should also be performed in conjunction with quantification of IL-36 protein levels and proteolytical activation.

The specific role of IL-36β, but not IL-36α and IL-36γ, in protection against lethal outcome of the HSV-1 infection (Fig. 2c) is intriguing. Using a vaginal model of HSV-1 infection, the lethal outcome of the disease in mice has recently been linked to dissemination of the virus to the enteric nervous system, resulting in inflammation mediated nerve damage, constipation and toxic megacolons^48^. In the HSV-1 flank skin infection model used here, moribund mice exhibit a gastro-intestinal phenotype (Fig. 3a) suggesting a similar loss of peristalsis leading to death. Furthermore, HSV-1 DNA can be detected in several vital organs such as the brain, liver and lungs (Fig. 3b). Interestingly, neurons can express IL-36β, and astrocytes and microglia have the receptor for IL-36, IL-1RL2^26, 27^. Hence, it is possible that IL-36β protects against lethal outcome of HSV-1 infection (Fig. 2c) by modulating the ability of the virus to spread and/or replicate in the nervous system. The cellular source(s) of IL-36 and induction pathways in the skin, and possibly the nervous system, remain to be determined. It is conceivable that the IL-36s act in local microenvironments, and thereby regulate very different physiological responses to infections. Further studies will be required to establish these mechanisms and functions.

The IL-36s promote neutrophil recruitment in different types of psoriasis^25, 41^. Neutrophils play an important role in guiding CD8^+^ cells to sites of influenza infection in the lungs^61^, and HSV-1 skin lesions are well known to be rich in neutrophils (see Wojtasiak *et al*.^62^ for refs). However, intradermal administration of IL-36α does not promote recruitment of CD8^+^ cells^39^, and removal of neutrophils does not affect the outcomes of HSV-1 skin infection^62^. Hence, neutrophils may have distinct anti-viral functions in the lungs that are not observed in the skin. While this manuscript was being prepared, it was reported that mice deficient in the receptor for IL-36, IL-1RL2 (also known as IL-36R), are protected from the lethal outcome of lung influenza virus infection^20^. The reduced mortality was associated with decreased lung inflammation, e.g., neutrophil infiltration, and tissue damage^20^. Furthermore, the virus induced inflammation was IL-36α dependent^20^. The latter is in agreement with our studies in the imiquimod skin inflammation model, where neutrophils are recruited in response to increased IL-36α expression^41^. However, the former is the opposite outcome, compared to the increased mortality seen here in the HSV-1 infection model in the absence of IL-36β signaling (Fig. 2c). The distinct phenotype in the HSV model used here may be linked to a unique role of IL-36β in the nervous system as discussed above. However, IL-36β also appears to have a distinct role in the skin. If the observed skin lesions (Figs. 2b and 7) were caused by inflammation induced by IL-36β, we would expect IL-36β KO mice to have smaller lesions than wild type; however, larger lesions are in fact observed (Fig. 7). *In vitro* studies with keratinocytes may reveal if IL-36β can modulate innate immunity in these cells. Additional studies of other viruses may also determine if these are functions specific to HSV or neurotropic pathogens, or if they represent universal protective mechanisms.

IL-36γ was shown recently to promote wound healing in the gut in experimental models of inflammatory bowel disease^63, 64^. IL-36 could have a similar function in the skin during HSV-1 infection. Such a wound healing property could explain, at least in part, why lesions are larger in IL-36β KO mice than wild type (Fig. 7), and is supported by the extended expression of IL-36 at day 7 post-infection (Fig. 9a–c), when levels of virus are already declining (Figs 6 and 9d). It is also conceivable that IL-36 expression (Fig. 9a–c) is induced by wound healing responses and not the infection *per se*. This could explain the paradox that the IL-36β mRNA is induced *in vivo* (Fig. 9b), but not during an *in vitro* infection (Fig. 10b). However, since IL-36β deficient lesions progress to become larger (Fig. 7), not merely heal poorly, IL-36β must have additional activity aimed at restricting the virus or lesion progression. This could involve an innate immune mechanism that restricts the ability of HSV-1 to spread laterally within the proliferating layer of keratinocytes in the skin. Further studies of how IL-36β controls HSV-1 infection, and IL-36 promote wound healing will be needed to determine the specific mechanism(s) involved.

In summary, IL-36β clearly plays a critical role in controlling the outcome of HSV-1 infection (Figs. 2 and 7); however, further studies will be necessary to define the mechanisms whereby IL-36β acts, and how it is activated and released from cells.

## Methods

### Virus and viral titers

Dr. Harvey M. Friedman (University of Pennsylvania, Philadelphia, PA) kindly provided the clinical HSV-1 isolate NS^65^. Virus was propagated in Vero cells obtained from ATCC, Manassas, VA. Viral titers were determined by plaque assays as follows: Virus was diluted in 10-fold serial dilutions in DMEM serum-free medium (Invitrogen, Carlsbad, CA). Vero cells grown in a 12-well plate were washed with PBS immediately before infection. Cells were incubated with 200 μl viral dilutions for 1 hour at 37 °C with gentle rocking of the plate every 10 minutes. After infection, cells were washed with PBS and overlayed with a 1:1 mixture of 1.2% low melting temperature agarose and 2X medium (DMEM low-glucose (Sigma-Aldrich, St. Louis, MO), supplemented with 10% fetal bovine serum (FBS, Atlanta Biologicals, Lawrenceville, GA) and gentamicin (Invitrogen). Plaques were counted approximately 48 hours post-infection.

### Mice

Wild type C57BL/6 and RAG^−/−^ mice were obtained from the Jackson Laboratory, Bar Harbor, ME, and bred in-house. The IL-36α^−/−^, IL-36β^−/−^, and IL-36γ^−/−^ mice, originating from GlaxoSmithKline (NC), The Knockout Mouse Project (UC Davis, CA), and Mutant Mouse Regional Resource Center (University of North Carolina, NC), respectively, were recently described and have no apparent spontaneous phenotypes^41^. All strains were maintained on the C57BL/6 background. All procedures involving mice were approved by the Temple University Institutional Animal Care and Use Committee and in compliance with the U.S. Department of Health and Human Services Guide for the Care and Use of Laboratory Animals.

Mice were genotyped using ear punches processed as previously described^66^. The primers used were: Il1f6-forward, 5′ GTCACAGTTAAGGCGTTCACC 3′; Il1f6-wild-type-reverse, 5′ AAGGGCCAGGGCTACTCAC 3′; Il1f6-KO-reverse, 5′ CTTAATATGCGAAGTGGACCTG 3′; Il1f8-forward, 5′ CTTAGGGATTGCTGTCCTTG 3′; Il1f8-wild type reverse, 5′ GTGTTATGATTCGGTTCCCAC 3′; Il1f8-KO-reverse, 5′ GATAGGTCACGTTGGTGTAG 3′; Il1f9-wild-type-forward, 5′ CTGGGCTATTTGTATCTTCA 3′; Il1f9-wild-type-reverse, 5′ CACACCTGCTGGTCCAAGTC 3′; Il1f9-KO-forward, 5′ GGCGGATTTCTGAGTTGGAG 3′; Il1f9-KO-reverse, 5′ GCAGCGCATCGCCTTCTATC 3′; Il1r1-forward, 5′ GAGTTACCCGAGGTCCAGTGG 3′; Il1r1-KO-reverse, 5′ GAATGGGCTGACCGCTTCCTCG 3′; and Il1r1-WT-reverse, 5′ CCGAAGAAGCTCACGTTGTCAAG 3′.

### *In vivo* HSV-1 infections

Male and female mice were bred in house in a pathogen-free animal facility and used for experiments. Mice were matched for age (7–12 weeks) and sex in each individual experiment. The infection protocol was obtained from Dr. Friedman^52^. Mice were denuded the day before infections by sequential shaving and epilating cream application. Cream was removed by rinsing with water. Scratch inoculations were performed with 1.5 × 10^6^ PFU HSV-1 on the right flank. Mice were photographed next to a ruler every second day. Image pixels were converted to mm using Image J (https//imagej.net/Welcome) and the depicted ruler. Skin lesions were outlined and sized in Image J. Blood was collected by cardiac puncture immediately following euthanasia and serum stored at −20 °C.

### Cell culture and treatments

Pooled human neonatal primary keratinocytes (Thermo Fisher Scientific, Carlsbad, CA) were maintained in Defined Keratinocyte Serum Free Medium supplemented with 50 μg/mL gentamicin (Invitrogen). HaCaT cells (provided by Dr. Meenhard Herlyn, Wistar Institute, Philadelphia, PA) were maintained in Dulbecco’s modified Eagle’s medium (Thermo Fisher Scientific) supplemented with 10% (vol./vol.) FBS and 50 μg/ml gentamicin. Cells were treated with cytokines as previously described^58^.

### Western blotting

HaCaT cells infected with HSV-1 were detached from growth area and loaded on a single-well NuPAGE 10% Bis-Tris gel (Thermo Fisher Scientific) using standard SDS-PAGE reducing conditions. Proteins were transferred to a PVDF membrane and approximately 2 mm wide strips cut from the top to the bottom. Sera from HSV-1 infected mice were diluted 1:250 in PBS, 1% BSA, 0.05% tween 20, and individually probed on a strip each overnight. Strips were washed and developed with anti-mouse Ig conjugated with horseradish peroxidase (GE Healthcare Life Sciences, Pittsburgh, PA) and enhanced chemiluminescence.

### HSV-1 genome copy numbers in tissue

Mouse tissue samples were immediately frozen on dry ice following euthanasia. Primary infection sites were collected using 4-mm biopsy punches (Miltex, York, PA). Tissue was ground into a powder over dry ice with autoclaved mortar and pestle. DNA was extracted from tissue using the Qiagen DNeasy Blood & Tissue kit according to manufacturer’s instructions (Qiagen, Valencia, CA). Viral genome copy numbers were determined using quantitative real-time PCR with 500 ng DNA per reaction and the following primer/probe set: 5′ CGACCAACTACCCCGAT 3′ (forward primer), 5′ CACTATGACGACAAACAAAATCAC 3′ (reverse primer), and VIC-CAGTTATCCTTAAGGTCTC-MGBNFQ (probe). TaqMan Gene Expression Master Mix (Applied Biosystems, Foster City, CA) was used for PCR amplification on an Applied Biosystems StepOnePlus Real-Time PCR System. A DNA standard was generated using the above primer pairs and an aliquot of NS. The generated PCR product was cloned into the pGEM-T Easy vector (Promega, Madison, WI) and a single cloned confirmed by full length sequencing. The plasmid was linearized with *Pst*I restriction enzyme and quantified by nanodrop, after which genome copy numbers/μl were calculated. Serial dilutions (10-fold) were used to generate a standard curve from 1 × 10^7^ copies to 1 copy.

### Cloning, expression and purification of recombinant HSV-gD protein

The HSV-1 gD gene sequence (GenBank accession number: SBS69688) was amplified from DNA isolated from infected tissue using the primers 5′ CATGGGGTCCGCGGCAAATATG 3′ and 5′ GAGGACGGCTGGTCGTCTTCC 3′. *Bam*HI and *Eco*RI restriction sites were added to the N and C terminal ends of the gD gene by PCR using the primer pair 5′ AGTAGGATCCCATGGGGTCCGCGGCAAATATG 3′ and 5′ GAAGGAATTCGAGGACGGCTGGTCGTCTTCC 3′. The restriction site flanked gD amplicon was directionally cloned into pHUTA vector^67^ and confirmed by full length sequencing. The recombinant vector was transformed into *E. coli* BL21 DE3 cells. Overnight cultures were re-inoculated into fresh LB broth (1:100) and incubated at 37 °C with shaking until an OD of 0.6 was reached. The culture was induced with 1 mM isopropyl β-D-thiogalactopyranoside for 5 h. Bacteria were harvested by centrifugation at 10,000 × *g* for 10 min, resuspended in PBS (pH 7.4) and analyzed for expression by Western blot using antibodies against 6x His tag on the recombinant protein (Anti his probe-HRP: Santa Cruz Biotechnology, Santa Cruz CA). Recombinant gD protein was purified under denaturing conditions by immobilized metal affinity chromatography using Ni^2+^-NTA slurry (Thermo Fisher Scientific) according to the manufacturer’s protocol. The purified protein was ascertained by SDS-PAGE, and quantified by BCA protein assay against known bovine serum albumin standards.

### HSV-1 glycoprotein D (gD) direct ELISA

Anti-HSV-1 gD titers were measured using 2-fold serial dilutions of sera by indirect ELISA. Microtiter plates were coated overnight with 50 µl of 100 µg/ml gD antigen solution in coating buffer (0.08 M sodium carbonate and 0.02 M sodium bicarbonate, pH-9.6). Coated plates were blocked with 3% bovine serum albumin in PBS at room temperature for 3 h. Serially diluted test sera were added to each well and incubated at room temperature for 2 h. Wells were washed three times with PBST (PBS + 0.5% Tween 20) and incubated with horse radish peroxidase conjugated polyvalent anti-mouse Ig antibody for 1 hour in darkness. Wells were developed with 2,2′-azino-bis(3-ethylbenzothiazoline-6-sulphonic acid) substrate. OD values were measured at 492 nm, and end-point titers determined as the highest antibody dilution whose mean O.D. was at least twice more than the mean O.D. value of the negative control (PBS).

### Flow cytometry

Spleen and the draining inguinal lymph node were harvested from euthanized mice 6 days post-infection. Organs were pushed through 70 um cell strainers (one strainer per organ per mouse), into a 50 ml tube and washed with PBS. Spleen cells were treated with RBC lysis buffer (eBiosciences, Waltham, MA). Cells were resuspended in 5% FBS/PBS and counted by hemacytometer. Cells (1 × 10^6^) from each organ was plated into a 96-well conical bottom plate and spun down. Cells were washed 2 times with 0.1% BSA/PBS. Cell pellets were incubated with a 1/40 dilution R-PE labeled H-2Kb SSIEFARL Pro5 MHC Pentamer (ProImmune, F188-82A-D, Sarasota, FL), for 10 minutes at room temperature in the dark. Cells were washed 3 times with 0.1% BSA/PBS, then incubated with cocktail containing 1/80 dilution APC-CD3 (eBiosciences anti-mouse CDS APC clone: 17A2), 1/200 dilution PeCy7-CD4 (BD pharmingen rat anti-mouse CD4 clone:RM4-5, San Jose, CA) and 1/100 dilution FITC-CD8 (ProImmune rat anti-mouse CD8 FITC (KT15) IgG2a), for 30 minutes at 4 °C in the dark. Cells were washed 3 times with 0.1% BSA/PBS and fixed in 2x paraformaldehyde diluted in 0.1% BSA/PBS.

Cells were evaluated on a BD FACSCanto utilizing FACSDiva software. 1 × 10^5^ events were collected for each sample gated on primarily live cells using the forward scatter channel (FSC). FlowJo v:10.1r5 was used to analyze data. Samples were gated for total cell populations (lymphocytes, granulocytes and antigen presenting cells) based on side scatter chanel (SSC) vs FSC comparisons on previously ungated cells. Sample analysis of CD3^+^, CD4^+^, CD8^+^ populations were gated on singlet cells via SSC-W vs SSC-H, followed by further gating on lymphocytes via SSC vs FSC comparisons. PerCP-Cy5.5 was utilized as a dump channel to reduce background fluorescence in comparisons via PerCP-Cy5.5 vs APC comparisons. For total CD3^+^, CD4^+^, and CD8^+^ population comparisons, SSC was compared to APC, PeCy-7, and FITC respectively. Cell percentages were multiplied by total cells counted from organs by hemocytometer. For CD8^+^ pentamer^+^ analysis, CD3^+^ cells were gated in SSC vs APC-CD3 comparisons. Within the CD3^+^ cell gate, CD4^+^ and CD8^+^ cells were evaluated via PE-Cy7 vs FITC comparisons. CD8^+^ cells were gated and gB(498-505) (SSIEFARL) specificity was evaluated via PE-pentamer vs CD8^+^-FITC comparisons.

### ELISpot assays

Skin was harvested from euthanized mice 6 days post-infection. Primary lesions were excised with 8 mm biopsy punches (Miltex, York, PA). Upper and lower secondary lesions were combined per mouse. Skin was incubated, dermis-side down, in Dispase (1 U/ml, Stemcell, Vancouver, BC, Canada) at 37 °C for 90 minutes in a 24-well plate. Skins were then incubated in Trypsin/EDTA at 37 °C for 30 minutes. Skin was roughly homogenized with a scalpel blade and incubated in solution containing 1 mg/ml Collagenase A (Roche, Pleasanton, CA) with 40 ug/ml DNase I (Roche) at 37 °C for 1 hour. PBS with 10% heat inactivated FBS was added to each sample. Skin cells were isolated through a 100 μm cell strainer into a 50 ml tube and washed with PBS. Cells were pelleted and resuspended in RPMI (Hyclone GE, Logan, UT) with 10% FBS, 2 mM l-glutamine, 1% antibiotic-antimycotic (Thermo Fisher Scientific) and 50 μM β-mercaptoethanol (Sigma-Aldrich, St. Louis, MO). Cells (3.25 × 10^5^) were added to mouse CD4 + /IFN-γ specific ELISpot plate (R&D Systems, Minneapolis, MN) and processed as per manufacturer’s instructions. Cells were counted through visualization with a dissecting microscope.

### RNA isolation and expression analyses

RNA was isolated from mouse skin using RNeasy Plus Universal minikit with genomic DNA eliminator step (Qiagen) according to manufacturer’s instructions. Human RNA was isolated using RNeasy minikit (Qiagen) in a similar manner. 1 μg per sample RNA was reverse-transcribed for 1.5 hour using 1 μg oligo(dN)_6_, 5.5 U RNAguard Ribonuclease Inhibitor (GE Healthcare) and 10 U AMV reverse transcriptase (Promega, Madison, WI) according to manufacturer’s instructions. Inactivation of AMV was performed at 95 °C for 10 minutes. The relative gene expression was quantified using real-time PCR with the comparative ΔΔ*C*_t_ method utilizing either GAPDH or Actin as the housekeeping gene. For analyses of gene expression in mouse skin, the following primers were used: Mouse GAPDH-Forward, 5′ CTTGTGCAGTGCCAGCC 3′; Mouse GAPDH-Reverse, 5′ GCCCAATACGGCCAAATCC 3′; Mouse IL1F8-Forward, 5′ CACTATGCATGGATCCTCAC 3′; Mouse IL1F8-Reverse, 5′ TGTCTCTACATGCTATCAAGC 3′; Mouse IL1F6-Forward, 5′ GACCAGACGCTCATAGCAG 3′; Mouse IL1F6-Reverse, 5′ CTTTAGCACACATCAGGCAG 3′; Mouse IL1F9-Forward, 5′ ATGGACACCCTACTTTGCTG 3′; Mouse IL1F9-Reverse, 5′ CAGGGTGGTGGTACAAATC 3′; Mouse Actin-Forward, 5′ CACCAGTTCGCCATGGAT 3′; Mouse Actin-Reverse, 5′ CATCACACCCTGGTGCCTA 3′. HSV-1 gD mRNA levels were determined using the primers and probe for HSV-1 genome copy number determinations. Primers for human GAPDH, IL-36α, IL-36β and IL-36γ are listed elsewhere^22, 68^. Primers used to distinguish the two human IL-36β splice variants: IL1F8-F (common forward), 5′ TCTCTGTCTCTTCTGTGCAG 3′; IL1F8-RA (reverse isoform 1), 5′ GCTCTCTCTCACATCCAGG 3′; IL1F8-RB (reverse isoform 2), 5′ GGCTTCTGTGCTTTCTTCTC 3′.

### Computational and statistical analyses

Alignments were performed using Clustal omega tool from EMBL-EBI http://www.ebi.ac.uk/Tools/msa/clustalo/. Evolutionary analyses were conducted using the MEGA7 software http://www.megasoftware.net. The following sequences were used for protein alignments: hIL-36α, NP_055255.1; hIL-36β isoform 1, NP_055253.2; hIL-36β isoform 2, NP_775270.1; hIL-36γ isoform 1, NP_062564.1; hIL-36γ isoform 2, NP_001265497.1; mIL-36α, NP_062323.1; mIL-36β, NP_081439.1; mIL-36γ, NP_705731.2). All experiments were performed at least three times unless otherwise indicated. Statistical significance was calculated using student’s *t* tests unless stated otherwise. All data shown are arithmetic means ± standard deviations unless indicated otherwise.
